# *lncRNA-5583* Modulates Porcine Granulosa Cell Proliferation Through a *miR-193a-5p/ACVR1*-Associated Mechanism

**DOI:** 10.3390/ani16193159

**Published:** 2026-10-08

**Authors:** Jingyang Dong, Huapeng Zhang, Fan He, Qingchun Li, Mengfan Qi, Binbin Fu, Kun Yan, Yun Feng, Rong Zhou, Mengxun Li, Tao Huang

**Affiliations:** 1 College of Animal Science and Technology, Shihezi University, Shihezi 832003, China; 18899590672@163.com (J.D.); 13699358979@163.com (H.Z.); 18299075532@163.com (F.H.); 18899598025@163.com (Q.L.); qimengfan1998@sina.com (M.Q.); fbb15546093387@sina.com (B.F.); yankun@stu.shzu.edu.cn (K.Y.); 18785778959@163.com (Y.F.); 2 Institute of Animal Sciences, Chinese Academy of Agricultural Sciences, Beijing 100193, China

**Keywords:** *lncRNA-5583*, *miR-193a-5p*, ACVR1, sows, granulosa cells, ovarian follicle

## Abstract

Ovarian follicular development is essential for reproductive performance in sows, with granulosa cells playing a central role in follicular growth and function. This study investigated the function and regulatory mechanism of *lncRNA-ALDBSSCT0000005583* (*lncRNA-5583*), previously identified as differentially expressed in M2-stage follicles (5.0–6.9 mm in diameter) between Meishan and Duroc sows. Overexpression and loss-of-function experiments were performed in porcine granulosa cells, and candidate microRNA interactions and downstream targets were examined using bioinformatic prediction, dual-luciferase reporter assays, and molecular and functional analyses. Porcine granulosa cell experiments were performed using three independent pooled granulosa-cell isolations (*n* = 3). *LncRNA-5583* was identified as a 588-nucleotide non-coding RNA. Its overexpression promoted granulosa cell proliferation and reduced apoptosis, accompanied by corresponding changes in PCNA, BAX, Bcl-2, and the BAX/Bcl-2 ratio; *miR-193a-5p* overexpression attenuated these effects. LncRNA-5583 interacted with *miR-193a-5p* in a sequence-dependent manner, and *miR-193a-5p* directly targeted ACVR1. Inhibition of *miR-193a-5p* increased ACVR1 expression and was accompanied by increased Smad3 phosphorylation, whereas *lncRNA-5583* knockdown decreased both *ACVR1* expression and Smad3 phosphorylation. These findings support the involvement of the *lncRNA-5583/miR-193a-5p/ACVR1* regulatory axis in porcine granulosa cell proliferation.

## Simple Summary

Ovarian follicle development is essential for sow fertility and directly influences reproductive performance in pig production. Granulosa cells are important components of ovarian follicles and help support follicle growth and development. However, the molecular mechanisms controlling the growth and survival of these cells are not fully understood. In this study, we investigated the role of a long non-coding RNA called *lncRNA-5583* in porcine granulosa cells. Increasing the level of *lncRNA-5583* promoted granulosa cell proliferation and reduced cell death, whereas decreasing its level produced the opposite effects. Further experiments showed that *lncRNA-5583* interacts with a small regulatory RNA called *miR-193a-5p* and influences the expression of activin A receptor type 1, a gene involved in cell signaling. Changes in this regulatory relationship were also accompanied by changes in Smad3 phosphorylation. These findings improve our understanding of how non-coding RNAs regulate granulosa cell function and ovarian follicle development in pigs. This knowledge provides a basis for future studies investigating the relationship between non-coding RNA regulation, follicular function, and reproductive traits in pigs.

## 1. Introduction

Fertility is a crucial factor in the pig industry, as it directly impacts production efficiency and economic returns. Therefore, improving the reproductive capacity of sows is of great significance for the sustainable development of the pig industry [1,2]. Ovarian follicular growth and maturation are critical determinants of female fertility, and a higher ovulation rate is generally positively associated with an increased litter size [1,3]. As a major somatic cell population within ovarian follicles, granulosa cells play essential roles in follicular growth, oocyte maturation, ovulation, and steroid hormone production [4,5]. The balance between GC proliferation and apoptosis is closely associated with follicular growth and atresia [6]. Follicular development depends on coordinated interactions among oocytes, GCs, and theca cells [7,8,9], and signaling pathways of the TGF-β superfamily contribute to the regulation of GC proliferation, differentiation, and apoptosis [10,11,12]. Therefore, porcine GCs provide a relevant cellular model for investigating molecular mechanisms involved in the regulation of granulosa-cell function.

Long non-coding RNAs (lncRNAs) are transcripts longer than 200 nucleotides with limited protein-coding potential and can regulate gene expression through transcriptional and post-transcriptional mechanisms [13,14,15,16]. Some lncRNAs can also interact with microRNAs (miRNAs) and thereby influence miRNA-mediated regulation of target transcripts [17]. Increasing evidence indicates that lncRNAs participate in the regulation of ovarian GC function, including proliferation, apoptosis, and autophagy [18,19,20,21,22]. Several lncRNAs, such as *HOTAIR*, *NEAT1*, *FDNCR*, and *NORFA*, have been implicated in these processes [19,20,21,22]. However, the functional roles and regulatory mechanisms of many porcine ovarian lncRNAs remain insufficiently characterized.

In our previous transcriptomic study, *lncRNA-5583* was identified as a differentially expressed long non-coding RNA in M2-stage follicles between Meishan and Duroc sows [23,24]. Our previous characterization further showed that *lncRNA-5583* is a 588-nucleotide non-coding transcript predominantly localized in the cytoplasm [24]. However, its function and regulatory relationships in porcine GCs remain unclear. Therefore, the present study investigated the effects of *lncRNA-5583* on porcine GC proliferation and apoptosis and examined its regulatory relationships with *miR-193a-5p* and ACVR1.

## 2. Materials and Methods

### 2.1. Samples

Porcine GCs were isolated from ovaries obtained from the Lvyuandakang Slaughterhouse in Shihezi City. Fresh ovaries were placed in pre-warmed physiological saline containing 100 IU/mL penicillin and 100 μg/mL streptomycin at 37 °C and transported to the laboratory within 2 h. Ovaries were collected on multiple independent occasions and randomly allocated into non-overlapping pools at each collection. GCs were independently isolated and cultured from each pool, and each independently prepared pooled GC isolation was considered one biological replicate. New batches of ovaries were collected for subsequent experiments after cells from a given collection had been used. Because individual animal identification was unavailable, the exact number of donor animals contributing to each ovarian pool could not be determined.

### 2.2. Culture of Porcine GCs

Follicular fluid containing GCs was aspirated from ovarian follicles using a 1 mL syringe and collected into a 15 mL centrifuge tube containing DMEM (Gibco, Thermo Fisher Scientific, Waltham, MA, USA). The cell suspension was centrifuged at 800 rpm for 3 min, and the supernatant was discarded. The cell pellet was resuspended in complete medium containing 10% fetal bovine serum (Gibco, Grand Island, NY, USA), seeded into 6-well plates, and cultured at 37 °C in a humidified atmosphere containing 5% CO_2_. Freshly isolated primary GCs were used without passaging throughout the study. The GC isolation procedure had previously been characterized by FSHR expression in our earlier study [24]; FSHR expression was not re-evaluated for each individual isolation.

### 2.3. Vector Construction

The full-length sequence of lncRNA-5583 (588 nucleotides), previously determined by rapid amplification of cDNA ends and reported in our previous study [24], was used to construct the lncRNA-5583 overexpression vector. The coding sequence of porcine ACVR1 (Ensembl gene ID: ENSSSCG00000015874) was used to construct the ACVR1 overexpression vector. The lncRNA-5583 and ACVR1 inserts were amplified using the gene-specific primers listed in Supplementary Table S1. PCR products were separated by 2% agarose gel electrophoresis, purified, digested with the corresponding restriction enzymes, and ligated into the pcDNA3.1(+) vector. Recombinant plasmids were verified by Sanger sequencing.

### 2.4. Cell Transfection

Porcine GCs were seeded in 6-well plates and transfected at 70–90% confluence using Lipofectamine 3000 (Thermo Fisher Scientific, Waltham, MA, USA). For plasmid transfection, 5 μg of plasmid DNA and 5 μL of transfection reagent were used per well. miRNA mimics, inhibitors, and siRNAs were transfected at a final concentration of 50 nM using 5 μL of transfection reagent per well. The corresponding empty pcDNA3.1 vector, mimic NC, inhibitor NC, or siRNA-NC was used as the negative control. RNA was collected 24 h after transfection for RT-qPCR, whereas protein was collected at 48 h for Western blotting. Flow-cytometric apoptosis and EdU assays were performed 24 h after transfection. Transfection efficiency was evaluated by RT-qPCR. The sequences of the siRNAs, miRNA mimics/inhibitors, and corresponding controls are provided in Supplementary Table S1.

### 2.5. Annexin V-FITC/PI Double Staining

Cell apoptosis was assessed using an Annexin V-FITC Apoptosis Detection Kit (Vazyme, Nanjing, China). At 24 h after transfection, GCs were harvested and centrifuged at 1000 rpm for 5 min. The cell pellet was washed with PBS and resuspended in 500 μL of 1× Annexin V binding buffer. Cells were then stained with 5 μL Annexin V-FITC and 5 μL propidium iodide (PI) in the dark. Apoptosis was analyzed by flow cytometry (Hangzhou POWCLIN Medical Technology Co., Ltd., Hangzhou, China), and the percentages of early and late apoptotic cells were quantified.

### 2.6. Real-Time Quantitative PCR (RT-qPCR)

Total RNA was isolated from GCs using TRIzol reagent (Invitrogen, Carlsbad, CA, USA). RNA concentration and purity were assessed spectrophotometrically, and samples with an OD260/OD280 ratio of 1.8–2.0 were used for subsequent analysis. RNA integrity was evaluated by electrophoresis on a 1% formaldehyde-denaturing agarose gel. A total of 500 ng RNA was reverse-transcribed into cDNA using the PrimeScript Reverse Transcriptase Kit (Takara Bio Inc., Kusatsu, Shiga, Japan). RT-qPCR was performed using ChamQ Universal SYBR qPCR Master Mix (Nanjing Vazyme Biotech Co., Ltd., Nanjing, China) on an FQD-96A Touch Real-Time PCR System (Hangzhou Bioer Technology Co., Ltd., Hangzhou, China) in a total reaction volume of 20 μL. No-template controls and no-reverse-transcription controls were included to monitor contamination, and amplification specificity was assessed by melt-curve analysis. GAPDH was used as the endogenous reference for mRNA and lncRNA expression, whereas U6 was used as the reference for miR-193a-5p expression. Relative expression levels were calculated using the 2^−ΔΔCt^ method. Primer sequences are provided in Supplementary Table S1.

### 2.7. Western Blot Analysis (WB)

GCs were washed with ice-cold PBS and lysed in RIPA lysis buffer (Beijing Solarbio Science & Technology Co., Ltd., Beijing, China) supplemented with protease and phosphatase inhibitors for 30 min on ice. Equal amounts of protein were separated by 12.5% SDS-PAGE and transferred onto polyvinylidene difluoride membranes (Millipore, Darmstadt, Germany). The membranes were blocked with 5% skim milk and incubated overnight at 4 °C with the following primary antibodies: BAX (mouse, 1:2000, Proteintech, Cat. No. 60267-1-Ig), Bcl-2 (rabbit, 1:1000, Proteintech, Cat. No. 68103-1-Ig), PCNA (mouse, 1:1000, Proteintech, Cat. No. 60097-1-Ig), β-actin (rabbit, 1:1000, Proteintech, Cat. No. 81115-1-RR), ACVR1 (mouse, 1:1000, Proteintech, Cat. No. 11108-1-AP), Smad3 (rabbit, 1:1000, Affinity, Cat. No. AF6362), and phospho-Smad3 (p-Smad3; rabbit, 1:1000, BIOSS, Cat. No. bs-3425R). After washing, the membranes were incubated for 1 h at room temperature with DyLight 680-conjugated anti-rabbit IgG (H + L) (Cell Signaling Technology, Cat. No. 5366) or DyLight 800 4X PEG-conjugated anti-mouse IgG (H + L) (Cell Signaling Technology, Cat. No. 5257), as appropriate. β-actin was used as the loading control. Fluorescent signals were detected using an Odyssey CLx imaging system (LI-COR Biosciences, Lincoln, NE, USA), and band intensities were quantified by densitometric analysis. Smad3 phosphorylation was quantified as the p-Smad3/Smad3 ratio.

### 2.8. 5-Ethynyl-2′-deoxyuridine (EdU) Cell Proliferation Assay

Cell proliferation was assessed using the BeyoClick™ EdU-594 Cell Proliferation Assay Kit (Beyotime Biotechnology, Shanghai, China) according to the manufacturer’s instructions. At 24 h after transfection, 1 mL of 20 μM EdU working solution was added to each well containing 2 mL of culture medium, and the cells were incubated for 2 h. GCs were then fixed with 4% paraformaldehyde for 30 min at room temperature, permeabilized with 0.5% Triton X-100 for 10 min, and counterstained with Hoechst 33342 for 10 min in the dark. Images were acquired using an inverted fluorescence microscope, and three randomly selected fields were analyzed for each sample. The proportion of EdU-positive cells was calculated as the number of EdU-positive cells divided by the total number of Hoechst-positive nuclei × 100%.

### 2.9. Enzyme-Linked Immunosorbent Assay (ELISA)

Estradiol (E2) and progesterone (P4) concentrations in culture supernatants were measured using commercial porcine ELISA kits (E2, MM-0480O2; P4, MM-1205O2; MLBio, Shanghai, China) according to the manufacturer’s instructions. Culture supernatants were diluted fivefold before measurement, and each sample was assayed in technical triplicate. The calibration ranges were 12.5–200 pmol/L for E2 and 150–2400 pmol/L for P4, with detection sensitivities of 8 and 80 pmol/L, respectively. Hormone concentrations were calculated from the corresponding calibration curves, and technical triplicates were averaged to obtain one value for each biological replicate before statistical analysis.

### 2.10. Dual-Luciferase Reporter Assay

Two putative miR-193a-5p binding sites within lncRNA-5583 were predicted using RNAhybrid. The corresponding lncRNA-5583 sequence was cloned into the psiCHECK™ reporter vector to generate the wild-type (WT) construct, while both predicted miR-193a-5p binding sites were simultaneously mutated to generate the mutant (MUT) construct. Similarly, WT and MUT reporter constructs containing the predicted miR-193a-5p binding region in the ACVR13′-UTR were generated.

293T cells were co-transfected with the WT or MUT reporter plasmid and either miR-193a-5p mimic or mimic NC, generating four experimental groups. Cells at 70–90% confluence were serum-starved for 2 h before transfection. After 6 h, the medium was replaced with DMEM containing 10% serum without antibiotics, and cells were cultured for an additional 42 h. Luciferase activities were then measured using the Dual-Luciferase^®^ Reporter Assay System (Promega Corporation, Madison, WI, USA) according to the manufacturer’s instructions. Briefly, cells were washed three times with PBS and lysed with 200 μL PLB for 30 min. Aliquots (10 μL) of cell lysate were sequentially mixed with 50 μL Luciferase Assay Buffer II and 50 μL Stop & Glo^®^ Substrate to measure firefly and Renilla luciferase activities, respectively. Relative luciferase activity was calculated as the ratio of Renilla to firefly luciferase activity. Each experiment was performed in triplicate.

### 2.11. Follicle Culture

Porcine ovaries were collected from commercially slaughtered pigs at Lvyuandakang Slaughterhouse in Shihezi City. After washing with PBS containing penicillin and streptomycin, intact antral follicles with an initial diameter of 3–5 mm were carefully dissected from the ovaries. The follicles were randomly allocated to four treatment groups: LV-NC, LV-lncRNA-5583, sh-NC, and sh-lncRNA-5583. Three follicles were cultured together in each well of a 24-well plate containing 1 mL of DMEM/F12 supplemented with 15% serum and penicillin–streptomycin at 38.5 °C in a humidified atmosphere containing 5% CO_2_.

For lentiviral treatment, the LV5 vector (EF-1αF/GFP&Puro) was used to construct the lncRNA-5583 overexpression lentivirus (LV-lncRNA-5583), whereas the LV3 vector (H1/GFP&Puro) was used to construct the lncRNA-5583 knockdown lentivirus (sh-lncRNA-5583). The corresponding LV-NC and sh-NC lentiviruses were used as negative controls. All lentiviral constructs were synthesized by Shanghai Jima Gene (Shanghai, China) at a titer of 1 × 10^8^ TU/mL. A total dose of 1 × 10^7^ TU was added to each well containing three follicles. At 12 h after lentiviral infection, the culture medium was replaced once with fresh medium and was not changed thereafter. Parallel wells were harvested at 24 h for RT-qPCR analysis of lncRNA-5583, BAX, Bcl-2, and PCNA expression, whereas separate parallel wells were harvested at 48 h for Western blot analysis of BAX, Bcl-2, and PCNA protein levels.

### 2.12. Bioinformatic Analysis

Candidate miRNAs potentially interacting with lncRNA-5583 were predicted using RNAhybrid and miRanda based on miRNA sequences obtained from miRBase 22. Nine evolutionarily conserved candidate miRNAs were identified across human, mouse, rat, cattle, and pig. Among these candidates, miR-193a-5p was prioritized for subsequent experimental analysis based on the bioinformatic predictions together with previous evidence of its potential relevance to ovarian biology. Candidate target genes of miR-193a-5p were independently predicted using ENCORI, TargetScan 7.1, and miRWalk. Genes shared among the three prediction datasets were retained, yielding 66 common candidate target genes for subsequent Gene Ontology (GO) and Kyoto Encyclopedia of Genes and Genomes (KEGG) enrichment analyses. The databases and bioinformatic resources used in this study, together with their corresponding website addresses, are provided in the Supplementary Materials. Complete candidate-miRNA and target-gene lists are also provided in the Supplementary Materials (Table S2–S10).

### 2.13. Statistical Analysis

Statistical analyses were performed using SPSS Statistics 17.0 (IBM, Armonk, NY, USA). For porcine GC experiments, each independently prepared pooled GC isolation was considered one biological replicate. For follicle-culture experiments, the three follicles cultured within one well constituted one experimental unit, and independently prepared follicle batches were considered biological replicates, and technical replicates from the same isolation were averaged before analysis. Comparisons between two matched groups were performed using paired two-tailed Student’s *t*-tests. Multiple-group comparisons were analyzed using repeated-measures one-way analysis of variance (ANOVA) followed by Šídák’s multiple-comparisons test, whereas two-factor experiments were analyzed using two-way ANOVA with the interaction term included. Results are expressed as mean ± standard error of the mean (SEM), and the statistical test and biological *n* are specified in the corresponding figure legends. Graphical representations were generated using GraphPad Prism 8.0 (GraphPad Software, San Diego, CA, USA). Statistical significance was defined as *p* < 0.05. * *p* < 0.05 and ** *p* < 0.01; ns, not significant.

## 3. Results

### 3.1. lncRNA-5583 Promotes the Proliferation of Porcine GCs

**Figure 1:**
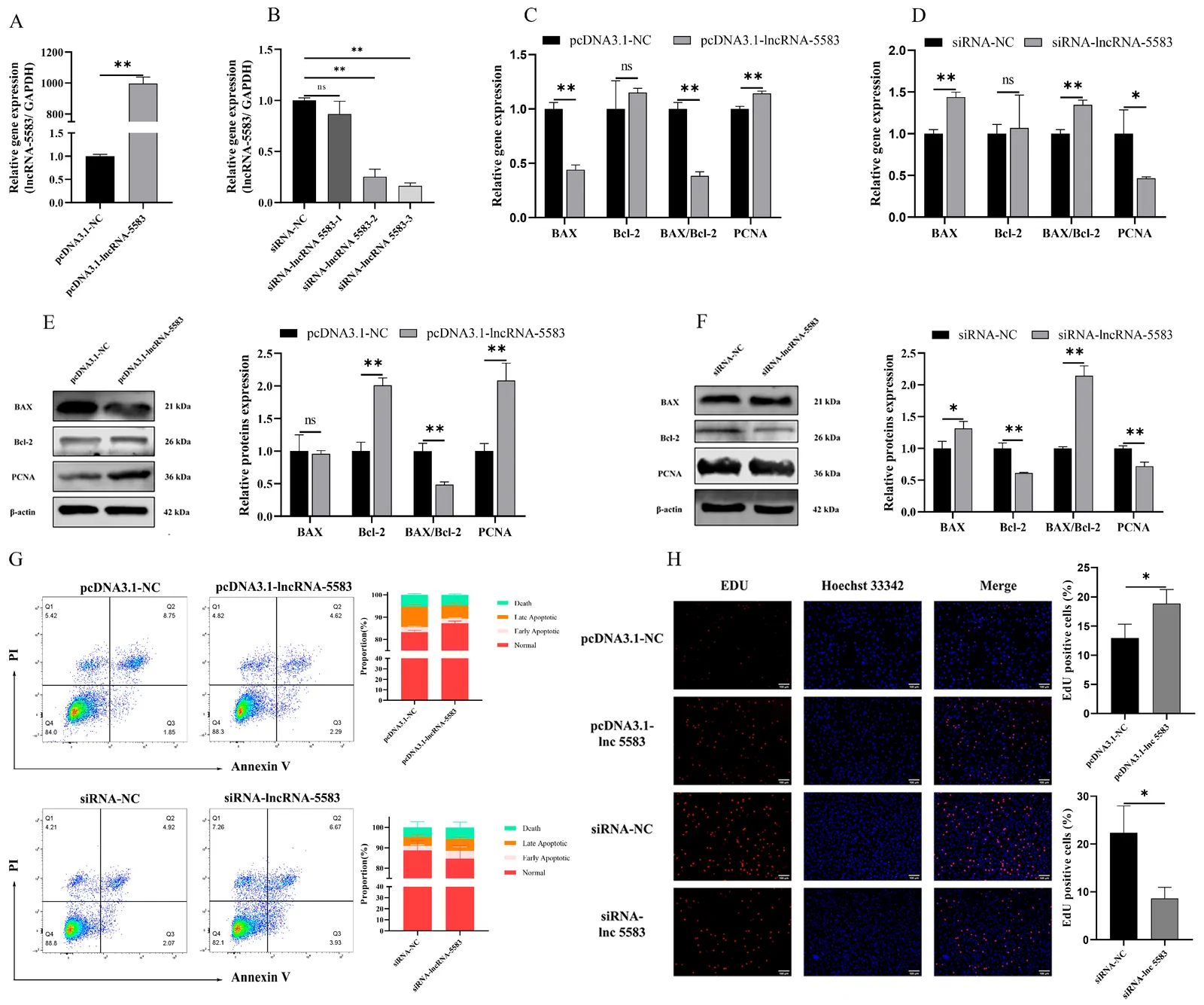
Effects of lncRNA-5583 on porcine GC proliferation and apoptosis. (**A**,**B**) Expression of *lncRNA-5583* in GCs following overexpression or knockdown. The *lncRNA-5583* overexpression group was transfected with the pcDNA3.1-lncRNA-5583 vector, and the corresponding negative control group was transfected with the empty pcDNA3.1 vector. The knockdown group was transfected with siRNA targeting *lncRNA-5583* (siRNA-lncRNA-5583), and the corresponding negative control group was transfected with nonspecific control *siRNA* (siRNA-NC). (**C**–**F**) mRNA (**C**,**D**) and protein (**E**,**F**) expression of apoptosis-related genes in GCs from each group. (**G**) Apoptosis of GCs assessed by flow cytometry. The colors in the flow cytometry plots represent event density, with red indicating the highest density, followed by yellow and green, and blue indicating the lowest density. (**H**) GC proliferation assessed by EdU incorporation. Data were analyzed using paired two-tailed Student’s *t*-tests and are presented as mean ± SEM from three independent pooled GC isolations (*n* = 3). The blot images shown are from the same independent experiments used for densitometric analysis. * *p* < 0.05, ** *p* < 0.01; ns, not significant. Scale bar = 100 µm.

To further investigate the effects of *lncRNA-5583* on GC proliferation and apoptosis, an *lncRNA-5583* overexpression vector and lncRNA-5583-specific siRNA were transfected into GCs, resulting in significant overexpression and knockdown of *lncRNA-5583*, respectively (Figure 1A,B). Regarding proliferation, *lncRNA-5583* overexpression significantly increased PCNA mRNA and protein levels, whereas *lncRNA-5583* knockdown significantly decreased PCNA mRNA and protein levels (Figure 1C–F). Consistently, the EdU assay showed that *lncRNA-5583* overexpression promoted GC proliferation, whereas its knockdown inhibited GC proliferation (Figure 1H). Regarding apoptosis-related measures, *lncRNA-5583* overexpression significantly decreased BAX mRNA expression and the BAX/Bcl-2 mRNA ratio, whereas Bcl-2 mRNA expression showed an increasing trend that did not reach statistical significance. Conversely, *lncRNA-5583* knockdown significantly increased BAX mRNA expression and the BAX/Bcl-2 mRNA ratio, while Bcl-2 mRNA expression was not significantly altered (Figure 1C,D). At the protein level, *lncRNA-5583* overexpression significantly increased Bcl-2 protein expression and decreased the BAX/Bcl-2 ratio, while BAX protein expression was not significantly altered. In contrast, *lncRNA-5583* knockdown increased BAX protein expression and the BAX/Bcl-2 ratio and decreased Bcl-2 protein expression (Figure 1E,F). Consistent with these changes, flow cytometric analysis showed that *lncRNA-5583* overexpression inhibited GC apoptosis, whereas its knockdown promoted apoptosis (Figure 1G). In addition, E2 and P4 levels in the culture medium of GCs were significantly increased following lncRNA-5583 overexpression, whereas *lncRNA-5583* knockdown did not significantly affect E2 or P4 levels (Supplementary Figure S1).

### 3.2. lncRNA-5583 Regulates Proliferation- and Apoptosis-Related Factors in Cultured Porcine Follicles

**Figure 2:**
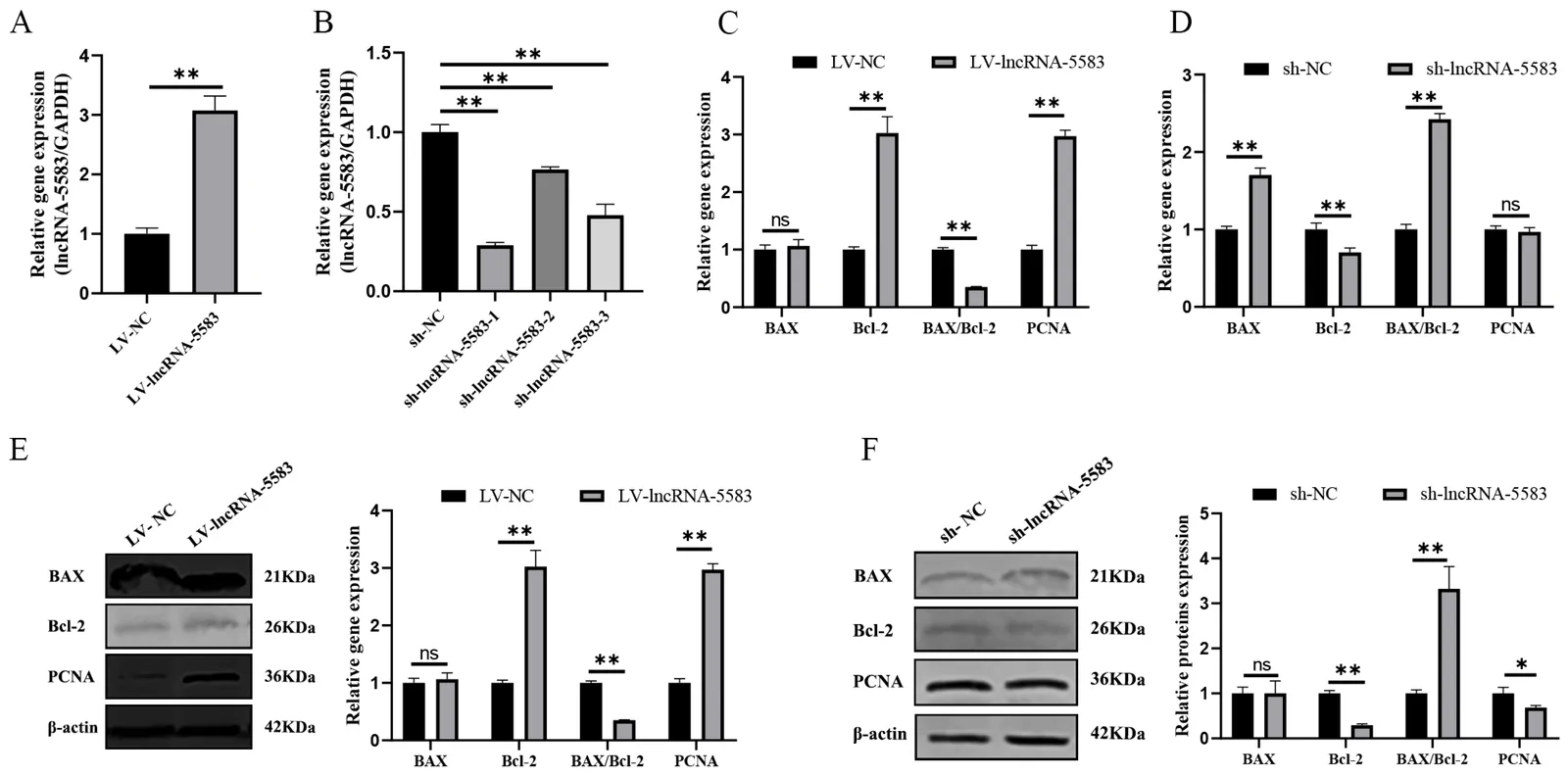
Effects of lncRNA-5583 on proliferation- and apoptosis-related factors in cultured porcine follicles. (**A**,**B**) The mRNA levels of *lncRNA-5583* in follicles. (**C**,**D**) The mRNA levels of *BAX*, *Bcl-2* and *PCNA* in follicles transfected with LV-lncRNA-5583 (**C**) and sh-lncRNA-5583 (**D**). (**E**,**F**) The protein levels of BAX, Bcl-2 and PCNA in follicles transfected with LV-lncRNA-5583 and sh-lncRNA-5583. Data were analyzed using paired two-tailed Student’s *t*-tests and are presented as mean ± SEM from three independent follicle culture experiments using independently prepared follicle batches (*n* = 3). * *p* < 0.05, ** *p* < 0.01; ns, not significant.

To investigate the effects of *lncRNA-5583* in cultured porcine follicles, isolated follicles were treated with LV-lncRNA-5583, LV-NC, sh-lncRNA-5583, or sh-NC. Representative morphological images of follicles at Day 0 and Day 2 are shown in Supplementary Figure S2. These images were used as representative morphological observations rather than as longitudinal measurements of individual follicle growth. RT-qPCR analysis showed that LV-lncRNA-5583 significantly increased *lncRNA-5583* expression, whereas sh-lncRNA-5583 significantly reduced its expression (Figure 2A,B). Additionally, LV-lncRNA-5583 significantly upregulated the mRNA expression of *Bcl-2* and *PCNA* while reducing the *BAX/Bcl-2* ratio (Figure 2C). In contrast, sh-lncRNA-5583 decreased *Bcl-2* and *PCNA* expression and increased the *BAX/Bcl-2* ratio (Figure 2D). Similar trends were observed at the protein level (Figure 2E,F).

### 3.3. lncRNA-5583 Interacts with miR-193a-5p in a Sequence-Dependent Manner

**Figure 3:**
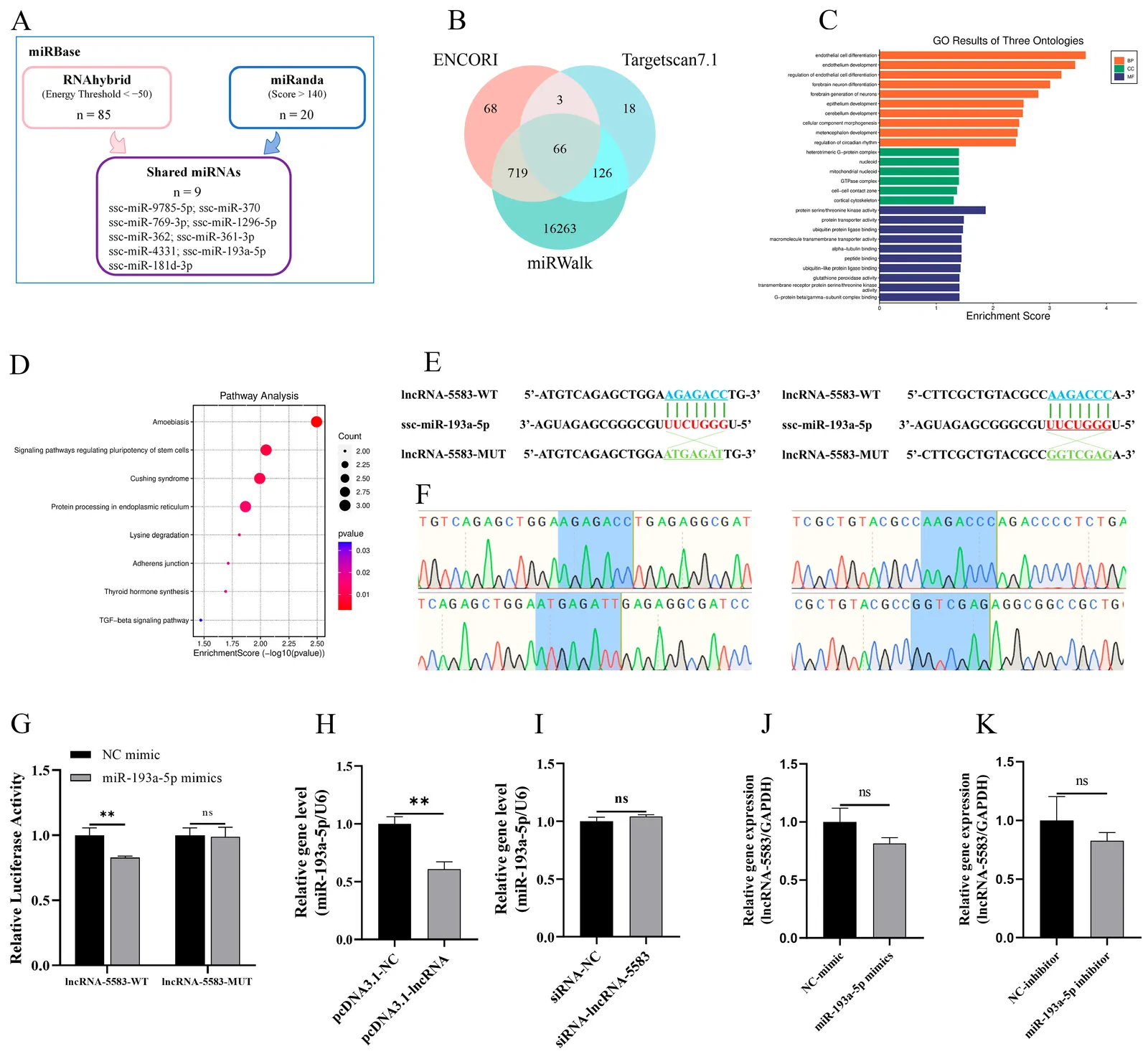
Interaction between lncRNA-5583 and miR-193a-5p. (**A**) miRNAs targeting *lncRNA-5583* predicted by RNAhybrid and miRanda. (**B**) Target genes of *miR-193a-5p* predicted by ENCORI, TargetScan7.1, and miRWalk. (**C**) GO analysis of *miR-193a-5p* target genes. (**D**) KEGG pathway analysis of *miR-193a-5p* target genes. (**E**) Schematic representation of the two predicted *miR-193a-5p* binding sites within wild-type *lncRNA-5583* (WT) and the corresponding mutant construct (MUT). In the MUT reporter, both predicted *miR-193a-5p* binding sites were simultaneously mutated. Red nucleotides indicate the *miR-193a-5p* seed-matching regions, and the mutated nucleotides are indicated in green. (**F**) Representative sequencing chromatograms confirming the wild-type and mutant *lncRNA-5583* reporter constructs. (**G**) Dual-luciferase reporter assay examining the interaction between *lncRNA-5583* and miR-193a-5p. (**H**,**I**) Expression of *miR-193a-5p* in GCs. (**J**,**K**) Expression of *lncRNA-5583* in GCs. Data in G were analyzed by two-way ANOVA followed by Šídák’s multiple-comparisons test; data in (**H**–**K**) were analyzed using paired two-tailed Student’s *t*-tests. Data are presented as mean ± SEM (*n* = 3 independent experiments for the luciferase assay; *n* = 3 independent pooled GC isolations for GC experiments).** *p* < 0.01; ns, not significant.

Previous characterization of *lncRNA-5583* showed that it is predominantly localized in the cytoplasm of porcine granulosa cells [24]. Based on this previously reported localization, we investigated its potential interaction with miRNAs. Utilizing the miRBase22 database, we predicted miRNAs potentially interacting with *lncRNA-5583* using RNAhybrid and miRanda algorithms, identifying nine evolutionarily conserved miRNAs across human, mouse, rat, cattle, and pig species (Figure 3A, Supplementary Table S6). Among these candidates, *miR-193a-5p* was prioritized for subsequent investigation based on the bioinformatic predictions and previous evidence implicating *miR-193a-5p* in ovarian development [25]. Candidate target genes of *miR-193a-5p* were further predicted using ENCORI, TargetScan 7.1, and miRWalk. The intersection of the three prediction datasets yielded 66 common candidate target genes (Figure 3B, Supplementary Table S10). GO enrichment analysis of these 66 genes identified enriched terms related to endothelial cell differentiation, regulation of endothelial cell development, cellular component organization, protein serine/threonine kinase activity, and transmembrane receptor protein serine/threonine kinase activity (Figure 3C). KEGG enrichment analysis identified several enriched pathways, including signaling pathways regulating pluripotency of stem cells, Cushing syndrome, lysine degradation, thyroid hormone synthesis, and TGF-β (Figure 3D).

RNAhybrid analysis identified two potential binding sites for *miR-193a-5p* and *lncRNA-5583* (Figure 3E). We constructed dual-luciferase reporter vectors of wild-type (WT) or mutant (MUT) *lncRNA-5583* fragment (Figure 3F). Dual-luciferase reporter assays demonstrated that overexpression of *miR-193a-5p* significantly suppressed the luciferase activity of the lncRNA-5583-WT reporter, whereas no significant change was observed in the lncRNA-5583-MUT group (Figure 3G). Furthermore, *lncRNA-5583* overexpression significantly reduced *miR-193a-5p* expression, whereas *lncRNA-5583* knockdown trended towards an increase in *miR-193a-5p* expression; however, this change was not statistically significant (Figure 3H,I). Contrarily, the modulation of *miR-193a-5p* levels through either overexpression or inhibition did not significantly affect *lncRNA-5583* expression in porcine GCs (Figure 3J,K). Together with the dual-luciferase reporter results, these findings support a functional interaction between *lncRNA-5583* and *miR-193a-5p* in porcine GCs.

### 3.4. miR-193a-5p Inhibits the Proliferation of GCs

**Figure 4:**
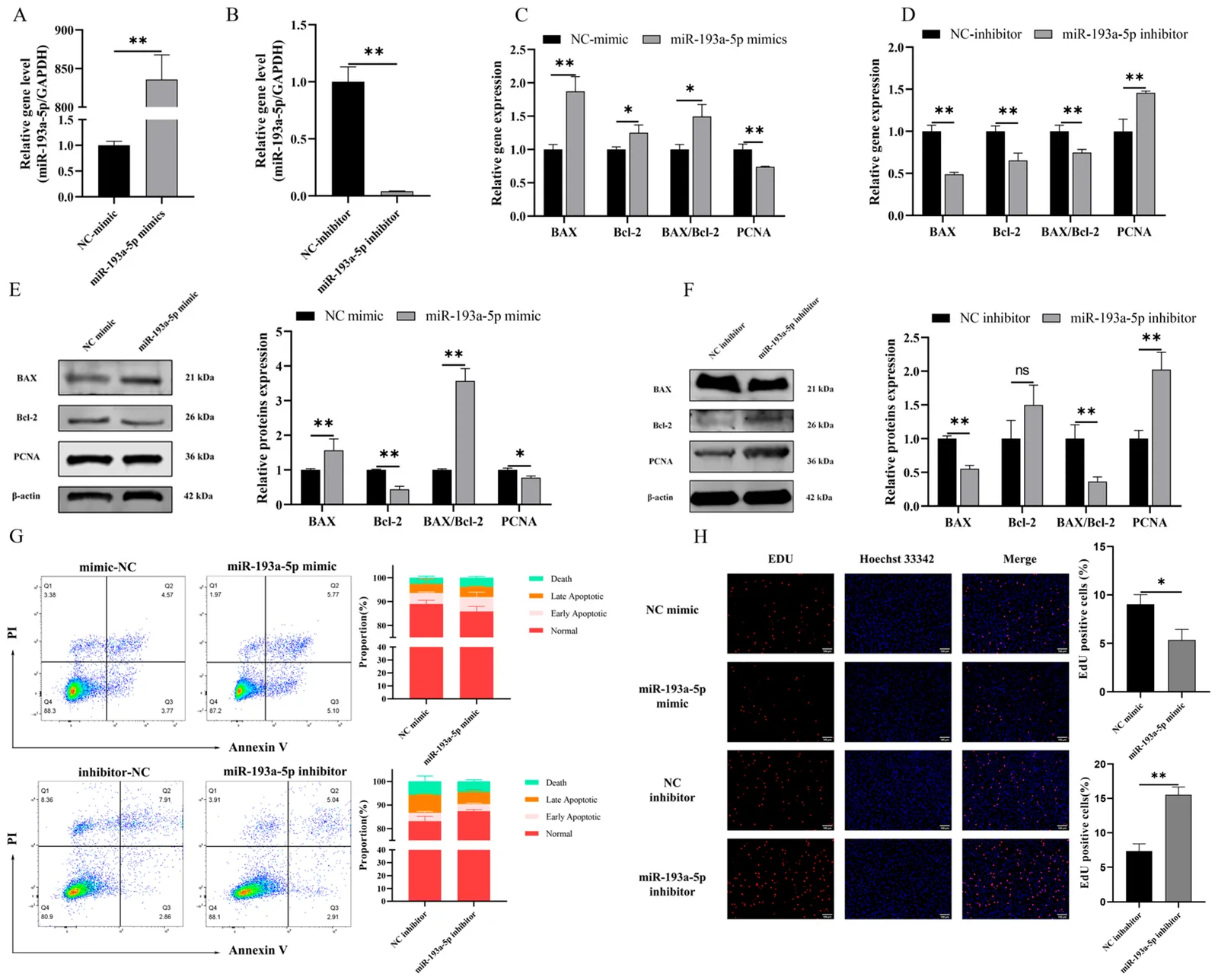
miR-193a-5p regulates the proliferation and apoptosis of GCs. (**A**,**B**) Expression of *miR-193a-5p* in GCs following transfection with the *miR-193a-5p* mimic or inhibitor. (**C**,**D**) mRNA expression of proliferation- and apoptosis-related markers in GCs. (**E**,**F**) Protein expression of proliferation- and apoptosis-related markers in GCs. (**G**) Apoptosis assessed by flow cytometry. The colors in the flow cytometry plots represent event density, with red indicating the highest density, followed by yellow and green, and blue indicating the lowest density. (**H**) Proliferation assessed by EdU incorporation. Data were analyzed using paired two-tailed Student’s *t*-tests and are presented as mean ± SEM from three independent pooled GC isolations (*n* = 3). * *p* < 0.05, ** *p* < 0.01; ns, not significant. Scale bar = 100 µm.

To further investigate the role of *miR-193a-5p* in porcine GCs, *miR-193a-5p* overexpression and inhibition models were established (Figure 4A,B). RT-qPCR analysis showed that, compared with the mimic NC group, *miR-193a-5p* mimic significantly increased *BAX* and *Bcl-2* mRNA expression as well as the *BAX/Bcl-2* ratio, while decreasing *PCNA* mRNA expression (Figure 4C). In contrast, *miR-193a-5p* inhibition significantly decreased *BAX* and *Bcl-2* mRNA expression and the *BAX/Bcl-2* ratio, whereas *PCNA* mRNA expression was increased (Figure 4D). Consistently, Western blot analysis showed that *miR-193a-5p* mimic significantly increased BAX protein expression and the BAX/Bcl-2 ratio, while suppressing Bcl-2 and PCNA protein expression (Figure 4E). Conversely, inhibition of *miR-193a-5p* significantly decreased BAX protein expression and the BAX/Bcl-2 ratio, increased PCNA protein expression, and slightly elevated Bcl-2 protein levels, although the change was not statistically significant (Figure 4F). Flow cytometric analysis showed that *miR-193a-5p* inhibition significantly reduced GC apoptosis (Figure 4G). EdU assays further demonstrated that *miR-193a-5p* mimic significantly suppressed GC proliferation, and *miR-193a-5p* inhibition produced the opposite effect (Figure 4H). Collectively, these results indicate that *miR-193a-5p* inhibits GC proliferation while promoting apoptosis in porcine granulosa cells.

### 3.5. miR-193a-5p Directly Targets ACVR1, Which Promotes Porcine Granulosa Cell Proliferation

**Figure 5:**
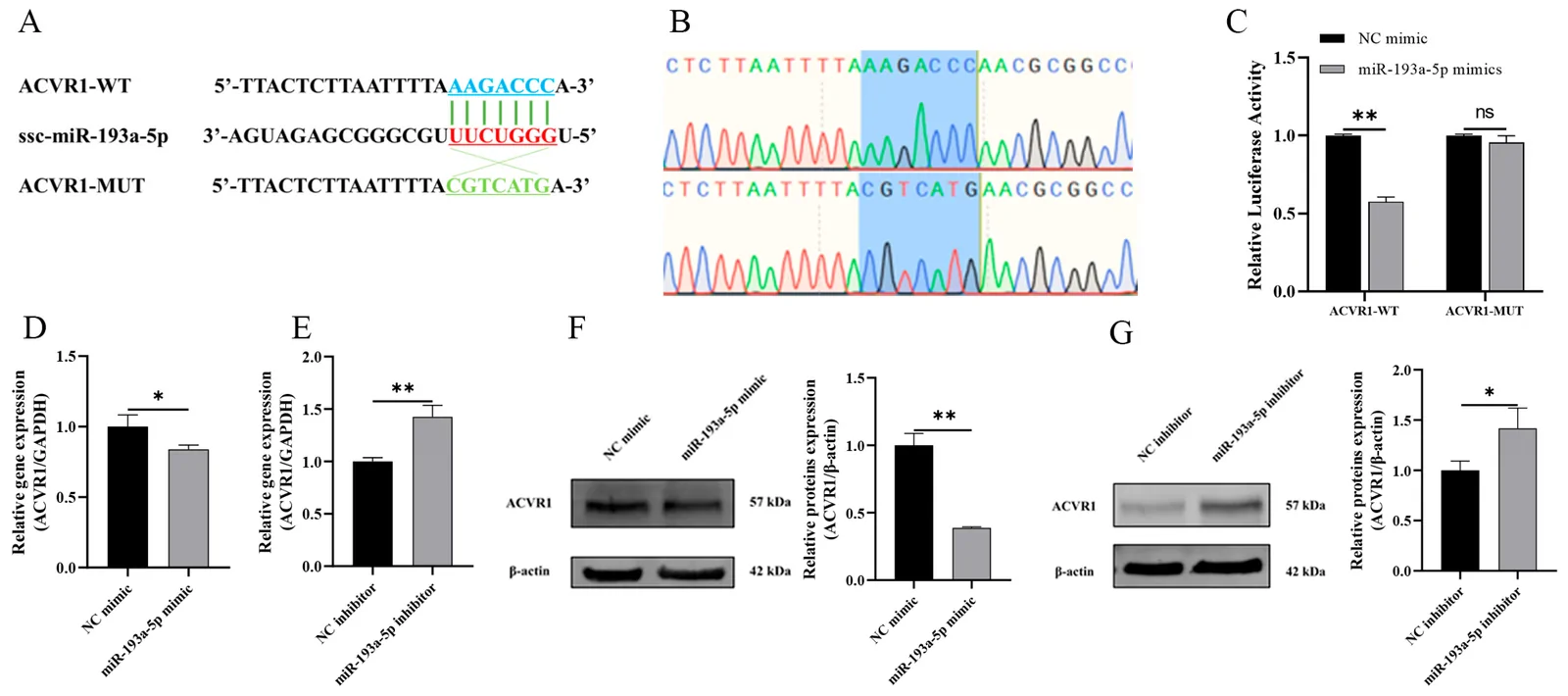
Targeted regulation of *ACVR1* by *miR-193a-5p*. (**A**) Schematic diagram predicting the interaction between *miR-193a-5p* and wild-type *ACVR1* (blue) or mutant *ACVR1* (green) using RNAhybrid. Red nucleotides indicate the seed sequence of *miR-193a-5p*. (**B**) Partial sequencing peak maps of the WT/MUT regions of the *ACVR1* dual-luciferase vector. (**C**) Dual-luciferase reporter assay of the miR-193a-5p–ACVR1 interaction. (D-G) Expression of *ACVR1* mRNA (**D**,**E**) and protein (**F**,**G**) detected in each group. Data in (**C**) were analyzed by two-way ANOVA followed by Šídák’s multiple-comparisons test; data in (**D**–**G**) were analyzed using paired two-tailed Student’s *t*-tests. Data are presented as mean ± SEM (*n* = 3 independent experiments for the luciferase assay; *n* = 3 independent pooled GC isolations for GC experiments). * *p* < 0.05, ** *p* < 0.01; ns, not significant.

Bioinformatic analysis of the predicted target genes of miR-193a-5p identified TGF-β among the enriched KEGG pathways (Figure 3D). Porcine *ACVR1* (Ensembl: ENSSSCG00000015874), also known as ALK2, is located on chromosome 15 and encodes a type I serine/threonine kinase receptor belonging to the TGF-β superfamily [26]. RNA hybrid analysis revealed that the seed sequence of *miR-193a-5p* perfectly complements the 3′-UTR of *ACVR1* (Figure 5A). Subsequently, we constructed dual-luciferase reporter vectors for ACVR1-WT and ACVR1-MUT (Figure 5B). Dual-luciferase assays demonstrated that co-transfection of ACVR1-WT with an *miR-193a-5p* mimic significantly reduced luciferase activity, whereas ACVR1-MUT exhibited no significant change (Figure 5C), thereby confirming the targeting relationship between *miR-193a-5p* and *ACVR1*. To further investigate whether *ACVR1* is regulated by *miR-193a-5p*, we observed that *miR-193a-5p* overexpression significantly decreased both *ACVR1* mRNA and protein levels, whereas *miR-193a-5p* inhibition significantly increased *ACVR1* expression (Figure 5D–G).

**Figure 6:**
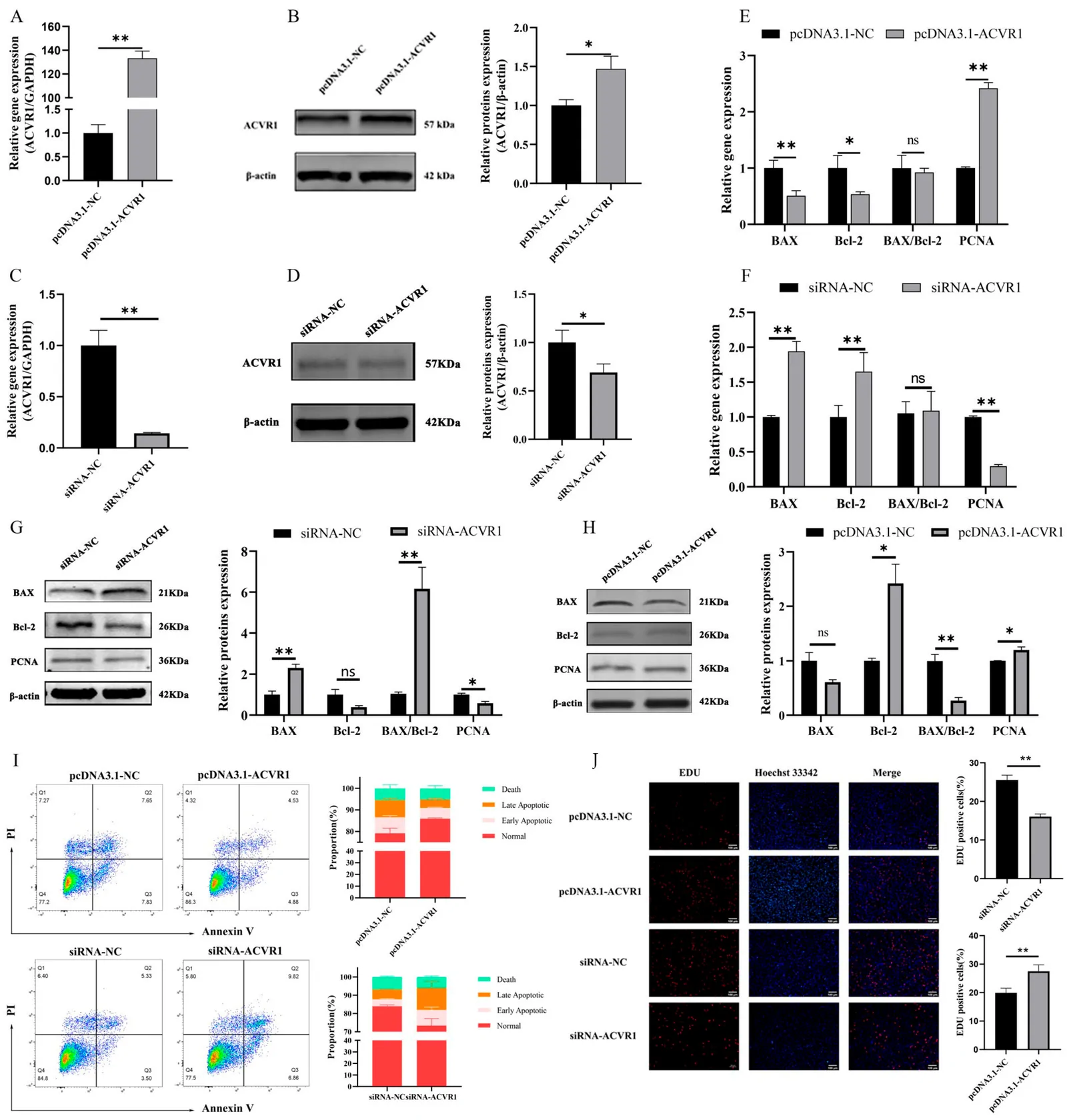
*ACVR1* regulates the proliferation and apoptosis of GCs. (**A**–**D**) *ACVR1* mRNA and protein expression following *ACVR1* overexpression or knockdown. (**E**,**F**) mRNA expression of apoptosis-related genes in GCs of each group. (**G**,**H**) Expression of proteins of apoptosis-related genes in granulosa cells of each group. (**I**,**J**) GC proliferation and apoptosis detected using flow cytometry (**I**) and EdU (**J**). The colors in the flow cytometry plots represent event density, with red indicating the highest density, followed by yellow and green, and blue indicating the lowest density. Data were analyzed using paired two-tailed Student’s *t*-tests and are presented as mean ± SEM from three independent pooled GC isolations (*n* = 3). * *p* < 0.05, ** *p* < 0.01; ns, not significant. Scale bar = 100 µm.

We investigated the role of *ACVR1* in GCs. We constructed *ACVR1* overexpression vector, pcDNA3.1-ACVR1, and siRNA-ACVR1 (Figure 6A–D). Overexpression of *ACVR1* significantly downregulated the mRNA expression of *BAX* and *Bcl-2*, although it did not significantly alter the *BAX/Bcl-2* expression ratio. Contrarily, the mRNA expression level of *PCNA* was significantly increased, whereas interference with *ACVR1* yielded the opposite results (Figure 6E,F). Additionally, the overexpression of *ACVR1* significantly downregulated the protein expression of BAX and the BAX/Bcl-2 ratio, and upregulated the protein level of PCNA, whereas the opposite trend was observed in the *ACVR1* knockdown group (Figure 6G,H). Flow cytometry further demonstrated that overexpression of *ACVR1* significantly inhibited GC apoptosis, whereas knockdown of *ACVR1* promoted it (Figure 6I). The percentage of EdU-positive cells was significantly increased by *ACVR1* overexpression and significantly decreased by *ACVR1* knockdown compared with their respective control groups (Figure 6J). Collectively, these results suggested that *ACVR1* promotes GC proliferation to a certain extent.

### 3.6. lncRNA-5583/miR-193a-5p/ACVR1 Axis Activates the TGF-β Pathway During GC Proliferation

**Figure 7:**
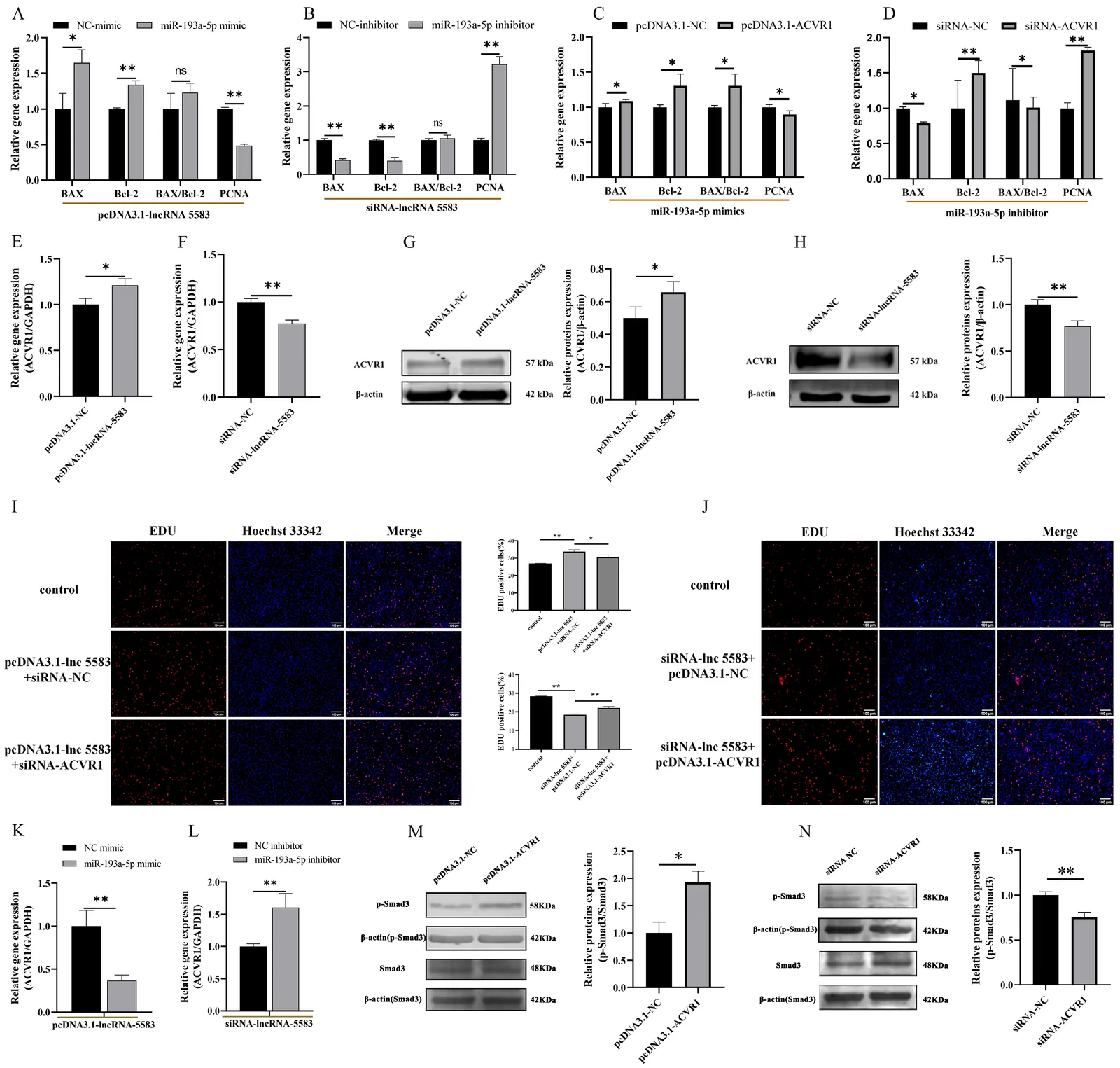
Regulatory relationship among *lncRNA-5583*, *miR-193a-5p*, and *ACVR1* in porcine GCs. (**A**–**D**) RT-qPCR was used to detect the mRNA expression of apoptosis-related genes in GCs of each group. (**E**,**F**) RT-qPCR was used to detect the mRNA expression of the *ACVR1* gene in GCs of each group. (**G**,**H**) Protein expression of the ACVR1 gene in GCs of each group. (**I**) EdU assay of GCs co-transfected with pcDNA3.1-lnc5583 and siRNA-NC or siRNA-ACVR1. (**J**) EdU assay of GCs co-transfected with siRNA-lnc5583 and pcDNA3.1-NC or pcDNA3.1-ACVR1. Scale bar = 100 μm. Control, cells co-transfected with empty pcDNA3.1 and siRNA-NC. (**K**,**L**) mRNA expression of the *ACVR1* gene in GCs of each group. (**M**,**N**) Protein expression of p-Smad3 and Smad3 in GCs of each group. Data are presented as mean ± SEM from three independent pooled GC isolations (*n* = 3). Data in A–D and I–L were analyzed using repeated-measures one-way ANOVA followed by Šídák’s multiple-comparisons test; data in (**E**–**H**) and (**M**–**N**) were analyzed using paired two-tailed Student’s *t*-tests. * *p* < 0.05, ** *p* < 0.01; ns, not significant.

To further investigate the functional relationship between *lncRNA-5583* and *miR-193a-5p* in porcine GCs, cells were co-transfected with the *lncRNA-5583* overexpression vector and either mimic NC or *miR-193a-5p* mimic, or with siRNA-lncRNA-5583 and either inhibitor NC or *miR-193a-5p* inhibitor. *miR-193a-5p* mimics reduced the expression of proliferation-related genes induced by *lncRNA-5583* overexpression, whereas *miR-193a-5p* inhibition attenuated the reduction in proliferation-related gene expression caused by siRNA-lncRNA-5583 (Figure 7A,B). These results support a functional regulatory relationship between *lncRNA-5583* and *miR-193a-5p* in the regulation of proliferation- and apoptosis-related gene expression in porcine GCs.

To determine whether *miR-193a-5p* regulates proliferation-related gene expression in GCs through ACVR1, GCs were co-transfected with pcDNA3.1-ACVR1 plus *miR-193a-5p* mimic or mimic NC, or with *siRNA-ACVR1* plus *miR-193a-5p* inhibitor or inhibitor NC. The mimic attenuated the effects of *ACVR1* overexpression on proliferation-related gene expression, whereas the inhibitor reversed those of *ACVR1* knockdown (Figure 7C,D). Together with the direct interaction between miR-193a-5p and the ACVR1 3′-UTR demonstrated by the dual-luciferase reporter assay, these results support the regulation of ACVR1 by miR-193a-5p in porcine GCs. We next examined whether lncRNA-5583 was associated with the regulation of ACVR1 expression. Together with the dual-luciferase reporter assay results, these findings indicate that ACVR1 is a direct target of miR-193a-5p in porcine GCs. Furthermore, lnc-5583 overexpression significantly increased ACVR1 mRNA and protein levels, whereas lnc-5583 knockdown significantly decreased them (Figure 7E–H). In rescue experiments, *lncRNA-5583* overexpression significantly increased the proportion of EdU-positive GCs, and this effect was partially reversed by ACVR1 knockdown (Figure 7I). Conversely, *lncRNA-5583* knockdown significantly decreased the proportion of EdU-positive cells, and this decrease was partially restored by ACVR1 overexpression (Figure 7J). These results suggest that *lncRNA-5583* promotes GC proliferation, at least in part, by upregulating ACVR1. Furthermore, *miR-193a-5p* overexpression attenuated the increase in *ACVR1* mRNA expression induced by *lncRNA-5583* overexpression (Figure 7K). Conversely, the inhibition of *miR-193a-5p* rescued the siRNA-lncRNA-5583-mediated downregulation of *ACVR1* mRNA (Figure 7L). These findings support a regulatory relationship among *lncRNA-5583*, *miR-193a-5p*, and ACVR1 in porcine GCs.

We further examined whether modulation of ACVR1 was associated with changes in Smad3 phosphorylation. Western blot analysis showed that ACVR1 overexpression was accompanied by an increase in the p-Smad3/Smad3 ratio, whereas ACVR1 knockdown was accompanied by a decrease in the p-Smad3/Smad3 ratio (Figure 7M,N). These findings indicate that modulation of ACVR1 expression is associated with altered Smad3 phosphorylation in porcine GCs. However, these results do not establish a direct ACVR1-to-Smad3 signaling pathway. Collectively, these findings suggest that *lncRNA-5583* positively regulates *ACVR1*, a direct target of *miR-193a-5p*, in porcine GCs, and that altered ACVR1 expression is accompanied by changes in Smad3 phosphorylation. Whether *lncRNA-5583* affects Smad3 phosphorylation remains to be determined.

## 4. Discussion

Granulosa cells are major somatic components of ovarian follicles and play important roles in follicular growth and atresia [27,28]. In our previous transcriptomic studies, *lncRNA-5583* was identified as differentially expressed in M2-stage follicles between Meishan and Duroc sows [23,24]; The present study further investigated the functional role of *lncRNA-5583* in porcine GCs and its associated regulatory mechanism.

Previous studies have shown that several lncRNAs, including *IFFD*, *NORHA*, and *SDNOR*, are involved in the regulation of porcine GC proliferation or apoptosis [29,30,31,32]. In the present study, lncRNA-5583 overexpression promoted porcine GC proliferation and reduced apoptosis, whereas *lncRNA-5583* knockdown produced the opposite effects. Consistent changes in proliferation- and apoptosis-related molecular markers were also observed in cultured porcine follicles; however, these findings do not directly demonstrate altered follicular development. In addition, *lncRNA-5583* overexpression increased E2 and P4 secretion, whereas its knockdown had no significant effect on either hormone. The basis for this asymmetric response remains unclear and requires further investigation.

Interactions between lncRNAs and miRNAs have been implicated in the regulation of GC proliferation and apoptosis in several species [21,33,34,35]. In the present study, bioinformatic prediction identified two potential *miR-193a-5p* binding sites within *lncRNA-5583*, and the dual-luciferase reporter assay supported a sequence-dependent interaction between *lncRNA-5583* and miR-193a-5p. *miR-193a-5p* also directly targeted the 3′-UTR of *ACVR1*. Expression analyses and phenotype-level rescue experiments further supported a regulatory relationship among *lncRNA-5583*, *miR-193a-5p*, and *ACVR1* in porcine GCs. However, because AGO2-RIP or RNA pull-down assays were not performed, the endogenous mechanism underlying the *lncRNA-5583-miR-193a-5p* interaction remains to be established.

ACVR1 is a type I serine/threonine kinase receptor of the TGF-β superfamily. ACVR1 (ALK2) should be distinguished from ACVR1B (ALK4), because canonical ACVR1 signaling is generally associated with Smad1/5/9, whereas activin-mediated Smad2/3 signaling is more commonly mediated through ACVR1B together with type II receptors such as ACVR2A/B [36]. Bioinformatic enrichment analysis identified TGF-β signaling among the predicted pathways associated with *miR-193a-5p* target genes; however, these predictions were used for candidate prioritization rather than as evidence of pathway activation. Previous studies have implicated ACVR1 in ovarian and follicular function [37,38]. In the present study, *ACVR1* overexpression promoted porcine GC proliferation, whereas ACVR1 knockdown produced the opposite effect. *ACVR1* overexpression was also accompanied by an increased p-Smad3/Smad3 ratio, whereas *ACVR1* knockdown was accompanied by a decreased ratio. Because canonical ACVR1-associated Smad1/5/9 signaling, ligand and type II receptor dependence, and pathway-selective inhibition were not examined, these findings do not establish Smad3 as a direct downstream effector of ACVR1. The observed changes in Smad3 phosphorylation should therefore be interpreted as molecular changes associated with ACVR1 modulation. Further studies are required to determine the downstream signaling mechanism by which *ACVR1* regulates porcine GC proliferation.

Taken together, the present findings support a sequence-dependent interaction between *lncRNA-5583* and *miR-193a-5p*, direct targeting of *ACVR1* by *miR-193a-5p*, and a regulatory relationship among *lncRNA-5583*, *miR-193a-5p*, and *ACVR1* in porcine GC proliferation. Modulation of *ACVR1* expression was also associated with changes in Smad3 phosphorylation, although the downstream signaling relationship remains unresolved. Given that GC proliferation and apoptosis are closely associated with follicular growth and atresia, this regulatory relationship may provide a molecular basis for further investigation of porcine follicular function. Future in vivo studies are needed to determine whether variation in the *lncRNA-5583/miR-193a-5p/ACVR1* regulatory network is associated with follicular growth, ovulation-related traits, or reproductive performance. Such validation will be necessary before components of this regulatory network can be considered as potential molecular markers or regulatory targets in pig reproduction.

## 5. Conclusions

In conclusion, this study demonstrates that *lncRNA-5583* interacts with *miR-193a-5p* in a sequence-dependent manner and modulates the inhibitory effect of *miR-193a-5p* on *ACVR1* expression in porcine GCs. Our findings support a regulatory relationship among *lncRNA-5583*, *miR-193a-5p*, and *ACVR1* in porcine GC proliferation, accompanied by changes in Smad3 phosphorylation. These findings provide new insights into the lncRNA-mediated regulation of porcine GC function and its potential relevance to follicular development.

## Data Availability

The original contributions presented in this study are included in the article and Supplementary Materials. Further inquiries can be directed to the corresponding authors.

## Supplementary Materials

The following supporting information can be downloaded at: https://www.mdpi.com/article/10.3390/ani16193159/s1, Figure S1: Effects of lncRNA-5583 on E2 and P4 secretion by porcine GCs; Figure S2: Representative morphology of cultured porcine follicles following modulation of lncRNA-5583. Table S1: Primer sequences for RT-qPCR and vector construction; Table S2: List of Chinese and English Abbreviations; Table S3: List of Online Prediction Databases and Tools; Table S4: RNAhybrid; Table S5: miRanda; Table S6: RNAhybrid and miRanda; Table S7: Candidate target genes of miR-193a-5p predicted by the Encyclopedia of RNA Interactomes (ENCORI); Table S8: Candidate target genes of miR-193a-5p predicted by TargetScan 7.1.; Table S9: Candidate target genes of miR-193a-5p predicted by miRWalk; Table S10: Ommon candidate target genes of miR-193a-5p predicted by ENCORI, TargetScan 7.1, and miRWalk.

## References

[1] Liu J., Qi N., Xing W., Li M., Qian Y., Luo G., Yu S. (2022). The TGF-β/SMAD Signaling Pathway Prevents Follicular Atresia by Upregulating MORC2. Int. J. Mol. Sci..

[2] Mao H., Chen L., Bao R., Weng S., Wang M., Xu N., Qi L., Wang J. (2022). Mechanisms of Oogenesis-Related Long Non-Coding RNAs in Porcine Ovaries Treated with Recombinant Pig Follicle-Stimulating Hormone. Front. Vet. Sci..

[3] Liu Y., Li M., Bo X., Li T., Ma L., Zhai T., Huang T. (2018). Systematic Analysis of Long Non-Coding RNAs and mRNAs in the Ovaries of Duroc Pigs during Different Follicular Stages Using RNA Sequencing. Int. J. Mol. Sci..

[4] Fan H.-Y., Liu Z., Shimada M., Sterneck E., Johnson P.F., Hedrick S.M., Richards J.S. (2009). MAPK3/1 (ERK1/2) in Ovarian Granulosa Cells Are Essential for Female Fertility. Science.

[5] LaVoie H.A. (2017). Transcriptional Control of Genes Mediating Ovarian Follicular Growth, Differentiation, and Steroidogenesis in Pigs. Mol. Reprod. Dev..

[6] Matsuda F., Inoue N., Manabe N., Ohkura S. (2012). Follicular Growth and Atresia in Mammalian Ovaries: Regulation by Survival and Death of Granulosa Cells. J. Reprod. Dev..

[7] Guan H.-Y., Xia H.-X., Chen X.-Y., Wang L., Tang Z.-J., Zhang W. (2021). Toll-like Receptor 4 Inhibits Estradiol Secretion via NF-κB Signaling in Human Granulosa Cells. Front. Endocrinol..

[8] Zhang Y., Lei K., Wang Z., Zhao F., Xing J., Zhu S., Li Y., Li S., Ding W., Wei Q. (2025). OONO^−^ Reduces the Expression of Steroidogenic Genes and Proteins to Inhibit Estrogen Secretion in Porcine Ovarian Granulosa Cells. Anim. Adv..

[9] Orisaka M., Tajima K., Tsang B.K., Kotsuji F. (2009). Oocyte-Granulosa-Theca Cell Interactions during Preantral Follicular Development. J. Ovarian Res..

[10] Rimon-Dahari N., Yerushalmi-Heinemann L., Alyagor L., Dekel N., Piprek R.P. (2016). Ovarian Folliculogenesis. Molecular Mechanisms of Cell Differentiation in Gonad Development.

[11] Knight P.G., Glister C. (2006). TGF-β Superfamily Members and Ovarian Follicle Development. Reproduction.

[12] Kaivo-oja N., Jeffery L.A., Ritvos O., Mottershead D.G. (2006). Smad Signalling in the Ovary. Reprod. Biol. Endocrinol..

[13] Golicz A.A., Bhalla P.L., Singh M.B. (2018). lncRNAs in Plant and Animal Sexual Reproduction. Trends Plant Sci..

[14] Hu S., Wang X., Shan G. (2016). Insertion of an Alu Element in a lncRNA Leads to Primate-Specific Modulation of Alternative Splicing. Nat. Struct. Mol. Biol..

[15] Khalil A.M., Guttman M., Huarte M., Garber M., Raj A., Rivea Morales D., Thomas K., Presser A., Bernstein B.E., van Oudenaarden A. (2009). Many Human Large Intergenic Noncoding RNAs Associate with Chromatin-Modifying Complexes and Affect Gene Expression. Proc. Natl. Acad. Sci. USA.

[16] Lee J.T. (2012). Epigenetic Regulation by Long Noncoding RNAs. Science.

[17] Liu S.J., Dang H.X., Lim D.A., Feng F.Y., Maher C.A. (2021). Long Noncoding RNAs in Cancer Metastasis. Nat. Rev. Cancer.

[18] Tu J., Chen Y., Li Z., Yang H., Chen H., Yu Z. (2020). Long Non-Coding RNAs in Ovarian Granulosa Cells. J. Ovarian Res..

[19] Jiang B., Xue M., Xu D., Song J., Zhu S. (2020). Down-Regulated lncRNA HOTAIR Alleviates Polycystic Ovaries Syndrome in Rats by Reducing Expression of Insulin-like Growth Factor 1 via microRNA-130a. J. Cell Mol. Med..

[20] Liu Y.-X., Ke Y., Qiu P., Gao J., Deng G.-P. (2023). LncRNA NEAT1 Inhibits Apoptosis and Autophagy of Ovarian Granulosa Cells through miR-654/STC2-Mediated MAPK Signaling Pathway. Exp. Cell Res..

[21] Yao X., Gao X., Bao Y., El-Samahy M.A., Yang J., Wang Z., Li X., Zhang G., Zhang Y., Liu W. (2021). lncRNA FDNCR Promotes Apoptosis of Granulosa Cells by Targeting the miR-543-3p/DCN/TGF-β Signaling Pathway in Hu Sheep. Mol. Ther. Nucleic Acids.

[22] Du X., Liu L., Li Q., Zhang L., Pan Z., Li Q. (2020). NORFA, Long Intergenic Noncoding RNA, Maintains Sow Fertility by Inhibiting Granulosa Cell Death. Commun. Biol..

[23] Li M., Liu Y., Xie S., Ma L., Zhao Z., Gong H., Sun Y., Huang T. (2021). Transcriptome Analysis Reveals That Long Noncoding RNAs Contribute to Developmental Differences between Medium-Sized Ovarian Follicles of Meishan and Duroc Sows. Sci. Rep..

[24] Zhang H., Zhang Q., He F., Qi M., Fu B., Li Q., Li M., Ma L., Liu Y., Huang T. (2024). Cloning and Identification of Differentially Expressed lncRNAs in Follicles of Meishan Pigs and Duroc Pigs with Their Correlation Analysis with miRNAs. Sci. Agric. Sin..

[25] Wang Y., Li N., Zhao J., Dai C. (2022). MiR-193a-5p Serves as an Inhibitor in Ovarian Cancer Cells through RAB11A. Reprod. Toxicol..

[26] Li H., Gao J., Wang J., Zhao B., Ma X., Zhang B., Lv C., Qiao H., Wang Y., Qing S. (2024). Bta-miR-301a Targets *ACVR1* to Influence Cleavage Time and Blastocyst Formation Rate of Early Embryos in Cattle. Biol. Reprod..

[27] Wang H. (2025). The Role of Granulosa Cells in Oocyte Development and Aging: Mechanisms and Therapeutic Opportunities. Semin. Cell Dev. Biol..

[28] Liu Y., Guo X., Fan J., Xie C., Huang T., Fu Y., Zhou R. (2024). CREBRF Regulates Apoptosis and Estradiol via ISG15/ISGylation in Pig Granulosa Cells. Free Radic. Biol. Med..

[29] Zhou X., He Y., Pan X., Quan H., He B., Li Y., Bai G., Li N., Zhang Z., Zhang H. (2023). DNMT1-Mediated lncRNA IFFD Controls the Follicular Development via Targeting GLI1 by Sponging miR-370. Cell Death Differ..

[30] Li Y., Zhang Z., Wang S., Du X., Li Q. (2023). miR-423 Sponged by lncRNA NORHA Inhibits Granulosa Cell Apoptosis. J. Anim. Sci. Biotechnol..

[31] Huo Y., Li Q., Yang L., Li X., Sun C., Liu Y., Liu H., Pan Z., Li Q., Du X. (2023). SDNOR, a Novel Antioxidative lncRNA, Is Essential for Maintaining the Normal State and Function of Porcine Follicular Granulosa Cells. Antioxidants.

[32] Sheng W., Xu Z., Fu Z., Zhang J., Du X., Li Y., Li Q. (2025). Lnc2300 Mediates the Induction of Granulosa Cell Apoptosis by Oxidative Stress. Anim. Adv..

[33] Zhu H., Chen Y., Zhang Z. (2020). Downregulation of lncRNA ZFAS1 and Upregulation of microRNA-129 Repress Endocrine Disturbance, Increase Proliferation and Inhibit Apoptosis of Ovarian Granulosa Cells in Polycystic Ovarian Syndrome by Downregulating HMGB1. Genomics.

[34] Wang M., Wang Y., Yao W., Du X., Li Q. (2022). Lnc2300 Is a Cis-Acting Long Noncoding RNA of CYP11A1 in Ovarian Granulosa Cells. J. Cell Physiol..

[35] Han X., Pan Y., Fan J., Wang M., Wang L., Wang J., Afedo S.Y., Zhao L., Wang Y., Zhao T. (2023). LncRNA MEG3 Regulates ASK1/JNK Axis-Mediated Apoptosis and Autophagy via Sponging miR-23a in Granulosa Cells of Yak Tertiary Follicles. Cell. Signal..

[36] Olsen O.E., Sankar M., Elsaadi S., Hella H., Buene G., Darvekar S.R., Misund K., Katagiri T., Knaus P., Holien T. (2018). BMPR2 Inhibits Activin and BMP Signaling via Wild-Type ALK2. J. Cell Sci..

[37] Knapczyk-Stwora K., Grzesiak M., Witek P., Duda M., Koziorowski M., Slomczynska M. (2019). Neonatal Exposure to Agonists and Antagonists of Sex Steroid Receptors Affects AMH and FSH Plasma Level and Their Receptors Expression in the Adult Pig Ovary. Animals.

[38] Kevenaar M.E., Themmen A.P.N., van Kerkwijk A.J., Valkenburg O., Uitterlinden A.G., de Jong F.H., Laven J.S.E., Visser J.A. (2009). Variants in the ACVR1 Gene Are Associated with AMH Levels in Women with Polycystic Ovary Syndrome. Hum. Reprod..

