# Supplementary material for: lncRNA-5583 Modulates Porcine Granulosa Cell Proliferation Through a miR-193a-5p/ACVR1-Associated Mechanism
Source: Animals (Basel). 2026 Oct 8;16(19):3159. doi: 10.3390/ani16193159 (PMC13663436; doi:10.3390/ani16193159)
Supplement: Supplementary file 1 [file animals-16-03159-s001.zip › supplement figure.pdf]

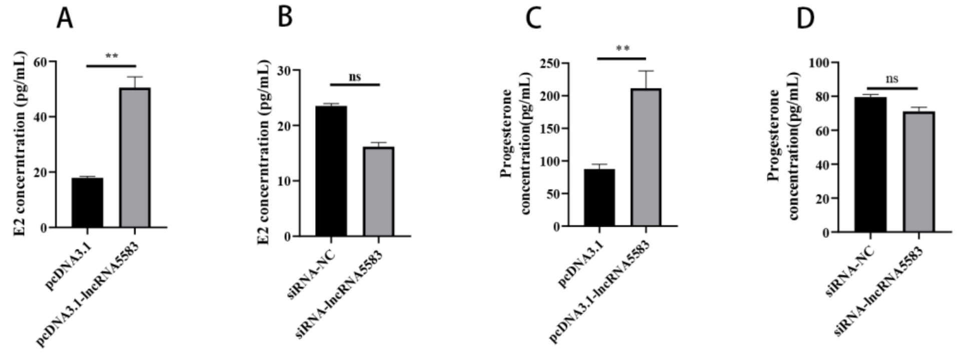

Figure S1. Effects of lncRNA-5583 on E2 and P4 secretion by porcine GCs. (**A**,**B**) E2 concentrations following lncRNA-5583 overexpression (**A**) or knockdown (**B**). (**C**,**D**) P4 concentrations following lncRNA-5583 overexpression (**C**) or knockdown (**D**). Data were analyzed using paired two-tailed Student’s *t*-tests and are presented as mean ± SEM from three independent pooled GC isolations (n = 3).P < 0.05, P < 0.01; ns, not significant.

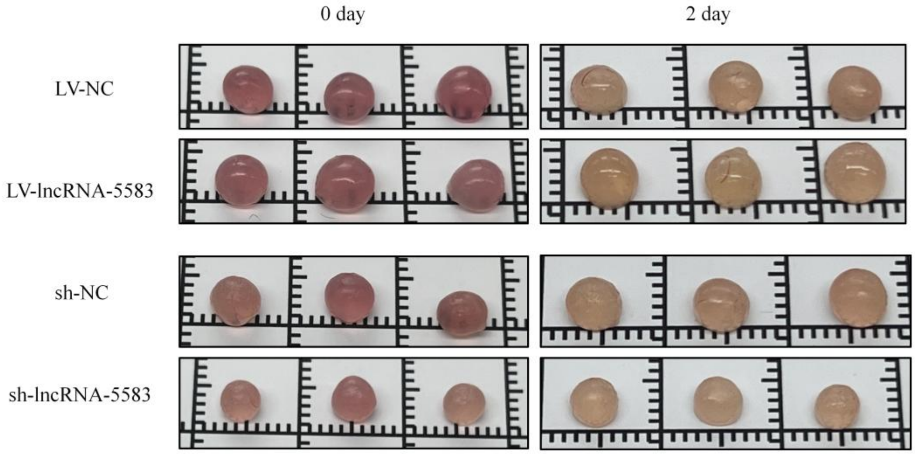

Figure S2. Representative morphology of cultured porcine follicles following modulation of lncRNA-5583. Representative images of follicles at Day 0 and Day 2 following treatment with LV-NC, LV-lncRNA-5583, sh-NC, or sh-lncRNA-5583. Three follicles were cultured per well. Because individual follicles within the same well could not be reliably matched between Day 0 and Day 2, the images are presented as representative morphological observations rather than longitudinal tracking of individual follicles.
